# Orbital Forcing of Martian Climate Revealed in a South Polar Outlier Ice Deposit

**DOI:** 10.1029/2021GL097450

**Published:** 2022-03-29

**Authors:** Michael M. Sori, Patricio Becerra, Jonathan Bapst, Shane Byrne, Riley A. McGlasson

**Affiliations:** ^1^ Department of Earth, Atmospheric, and Planetary Sciences Purdue University West Lafayette IN USA; ^2^ Physikalisches Institute Universität Bern Bern Switzerland; ^3^ Jet Propulsion Laboratory California Institute of Technology Pasadena CA USA; ^4^ Lunar and Planetary Laboratory University of Arizona Tucson AZ USA

**Keywords:** ice mounds, orbital forcing, craters, Burroughs, paleoclimate

## Abstract

Deciphering paleoclimate on Mars has been a driving goal of Martian science for decades. Most research has addressed this issue by studying Mars' large polar layered deposits (PLDs) as a paleoclimate proxy, but the certainty to which we know the link between climate and orbit is debated. Here, we instead consider the record of other, smaller ice deposits located within craters separated from the PLDs using images from NASA's High Resolution Imaging Science Experiment camera and signal processing techniques. We show that the climate record in Burroughs Crater (72.3°S, 116.6°E) contains robust evidence of orbital forcing, with periodicities that have wavelengths of 15.6 and 6.5 m. The ratio of these dominant wavelengths is 2.4, the same as the ratio between the periods of Mars' obliquity changes and orbital precession. This result suggests orbital control of recent Mars climate, and would imply an average ice accumulation rate of 0.13 mm/yr over 4.5 Myr in this region.

## Key Points

- The icy record in Burroughs crater shows Martian climate is strongly controlled by astronomical forcing
- The average ice accumulation rate in this region was 0.13 mm/yr
- The deposit in Burroughs crater represents a younger, more easily decipherable climate record than that in the SPLD

## 1 Introduction

The northern (NPLD) and southern (SPLD) polar layered deposits of Mars contain stratigraphic sequences of ice and dust that have been extensively studied as paleoclimate records (see review in Byrne, 2009). It has been hypothesized for decades that these records are controlled by Mars' orbital and rotational variations (e.g., Cutts & Lewis, 1982; Laskar et al., 2002; Murray et al., 1973; Smith et al., 2016), similar to Milankovitch cycles on Earth (Hays et al., 1976). Demonstrating such a link is one of the driving goals of Martian polar science (e.g., Smith et al., 2020), but statistically distinguishing this signal from stochastic variability has proven challenging. In particular, climate signals can resemble red noise, where longer wavelengths have greater power than shorter wavelengths and successive time samples are not completely random. Because of this issue, some previous authors (Perron & Huybers, 2009) have argued that compelling identification of orbital signals in Martian ice stratigraphy would require the robust detection of two periodic wavelengths that not only have a significant concentration of spectral energy, but also whose ratio corresponds to the ratio of two controlling orbital periodicities. Such an identification has been proposed in the sedimentary rock record of Mars (Lewis et al., 2008). Statistically significant wavelengths have been identified in the exposed layering of the NPLD (Becerra et al., 2017) and SPLD (Becerra et al., 2019), but they mostly have ratios that differ from orbital periodicities. The mismatch between ratios was attributed to large time‐variability of ice accumulation rates, but this explanation depends on the considered accumulation models, which often assume a priori that obliquity changes are the dominant control on polar ice accumulation rates (Hvidberg et al., 2012).

One path in advancing the field of Martian paleoclimate studies is to consider ice deposits other than the polar layered deposits (PLDs). Here, we consider ice deposits located within impact craters that are smaller than, and separate from, the PLDs. We argue that these crater deposits represent records in which orbital signals could be more straightforward to identify because they are at lower latitudes than the PLDs and thus might be more sensitive to obliquity variations. These deposits may also represent relatively young stratigraphy that has undergone fewer cycles of accumulation and ablation compared to the PLDs. Previous work has identified 18 crater ice deposits in the northern hemisphere (Conway et al., 2012) and 31 crater ice deposits in the southern hemisphere (Sori et al., 2019). High‐resolution images have been used to study surface morphology and constrain retreat rates of ice exposures in the northern deposits (Bapst et al., 2018; Brown et al., 2008). All the southern deposits are mantled with dust that mostly conceal their ice cores. Although subsurface radar sounding has been used to identify interior layering in one of the northern deposits (Brothers & Holt, 2016), the types of quantitative signal analyses that have been extensively used on exposed layering in the PLDs have not previously been applied to these crater ice deposits.

Here, we argue that these outlier ice deposits reveal orbital forcing of recent Martian climate. Our strategy is to read the record that is expressed in ancient layers exposed at the surface, an idea that has broadly been used to infer paleoclimate history from glaciers on Earth in, for example, Greenland (e.g., Schaefer et al., 2009) and Antarctica (e.g., Baggenstos et al., 2018). In Section 2, we describe how we constructed the paleoclimate proxy we used in our analysis: The morphological shape of layers visibly exposed in an ice deposit in Burroughs crater. In Section 3, we describe the signal analysis we performed on this paleoclimate proxy and the results. In Section 4, we discuss implications of these results for the climate of Mars and comparisons to the SPLD. We give concluding remarks in Section 5.

## 2 Construction of Paleoclimate Proxies at Burroughs Crater

**Figure 1:**
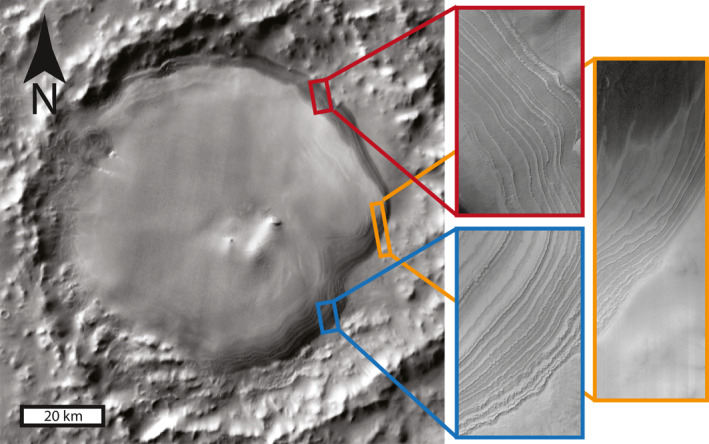
A layered ice deposit in Burroughs crater. Contextual image is from the Thermal Emission Imaging System instrument aboard the Mars Odyssey spacecraft, showing Burroughs crater and the interior ice deposit at 72°S, 117°E. North is up in this image. Insets are High Resolution Imaging Science Experiment (HiRISE) images showing widespread layer exposures. From top to bottom, the HiRISE image IDs are ESP_057848_1080, ESP_057439_1075, and ESP_058362_1070. These three HiRISE images are part of stereo pairs, from which high‐resolution digital elevation models were constructed.

We studied the 49 crater ice deposits inventoried by Conway et al. (2012) and Sori et al. (2019) and found that one had widespread exposed layering suitable for paleoclimate analysis. This ice deposit is located in Burroughs Crater (Figure 1), a 117‐km‐diameter impact crater located at 72°S, over 200 km away from the SPLD. The ice deposit is 74 km in diameter and has exposed, visible layering around most of its perimeter. The entire deposit has a few 100s of meters of topographic relief, with as much as ∼200 m of relief visible in some of the layer exposures. Although 12 other crater ice deposits in the southern hemisphere and 10 crater ice deposits in the northern hemisphere have some degree of visible layering, they all have little topographic relief, are obscured by dune cover or other superposed material, are only seen in very localized exposures, or a combination of the above (see Figure 1 in Supporting Information S1). Only the Burroughs deposit has the extensive, thick, unobscured, exposed visible stratigraphy necessary to obtain meaningful paleoclimate results. Based on the fact that some internal layering has been observed in subsurface radar sounding at other deposits (McGlasson et al., 2021), we speculate that the reason only Burroughs has extensive, thick exposures is related to regional atmospheric effects that locally enhance erosion along the perimeter of the deposit, and not because Burroughs is unique in its layered nature.

**Figure 2:**
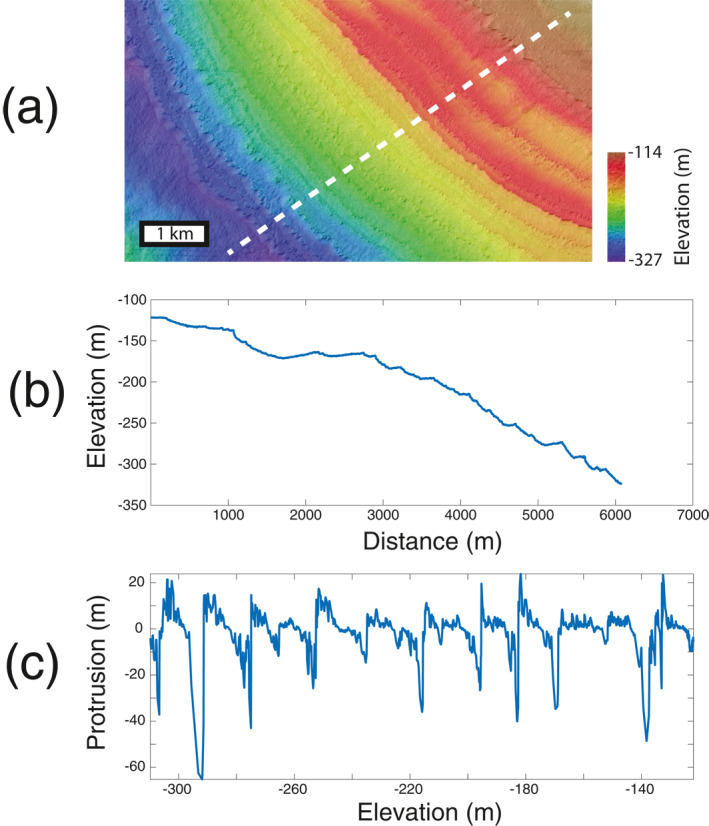
Ice deposit topography as a paleoclimate proxy. (a) High Resolution Imaging Science Experiment‐derived digital elevation model (DEM) DTEPC_058362_1070_057650_1070_A01, showing layer topography over ∼200 m of stratigraphic relief. (b) Typical topographic profile of layer topography, represented by dashed line in (a). (c) Layer protusion profile extracted from this DEM and used in our analysis.

We conducted a campaign to acquire high resolution images of Burroughs using the High Resolution Imaging Science Experiment (HiRISE, McEwen et al., 2007) aboard the Mars Reconnaissance Orbiter (MRO). In total, we obtained 18 HiRISE images of the Burroughs ice deposit. Of these 18 images, 6 images are stereo pairs of visible layering around the deposit's perimeter, from which we constructed 3 digital elevation models (DEMs). These 6 images were taken during southern summer (solar longitude ranging from 268° to 320°). The images have a resolution of ∼25 cm/pixel, while the DEMs can reliably resolve elevations of ∼1 m. The DEM that features the largest stratigraphic section of exposed layers (188 m) is shown in Figure 2a (ID: DTEPC_058362_1070_057650_1070_A01), while the other two DEMs (IDs: DTEPC_057439_1075_057241_1075_A01 and DTEPC_057716_1080_057,848_1,080_A01) are available on the HiRISE website. From these 3 DEMs, we derived stratigraphic profiles of layer protrusion.

Layer protrusion of icy stratigraphy on Mars has been shown to be a useful paleoclimate proxy. The degree to which icy layers on Mars protrude from a background slope is a reliable proxy for composition, with layers that protrude farther from a background slope being more dust‐rich and resistant to erosion (Becerra et al., 2016). This topographic metric may be more reflective of internal properties, like composition, than layer brightness (Herkenhoff et al., 2007). We constructed protrusion profiles in the following way. First, we extracted three topographic profiles from the HiRISE DEMs, 5 m apart and each extending from the top of the layered stratigraphy to its bottom, perpendicular to the layers. Second, for each profile, protrusion was calculated by using a moving window method. At each point along the topographic profile, protrusion was calculated by measuring the normal distance between the topography and the linear fit of the topography at the center of the window. We selected a window size of 350 m horizontally, based on the fact that is has been shown to be a reliable size for polar stratigraphy (Becerra et al., 2016) and that this window size captures the apparent topographic relief seen in layers of the ice deposit (e.g., Figure 2b). Finally, we averaged the three protrusion profiles together to reduce any noise introduced by local roughness variations not dependent upon internal layer properties (although in practice, we found that this averaging did not significantly affect our results because the topographic and protrusion profiles were sufficiently similar, Figure 2 in Supporting Information S1). This process was repeated for each of the three DEMs at Burroughs. It has been shown that this method yields robust paleoclimate proxies for icy stratigraphy on Mars (Becerra et al., 2016, 2017; 2019). An example protrusion profile is shown in Figure 2b.

## 3 Paleoclimate Analysis

**Figure 3:**
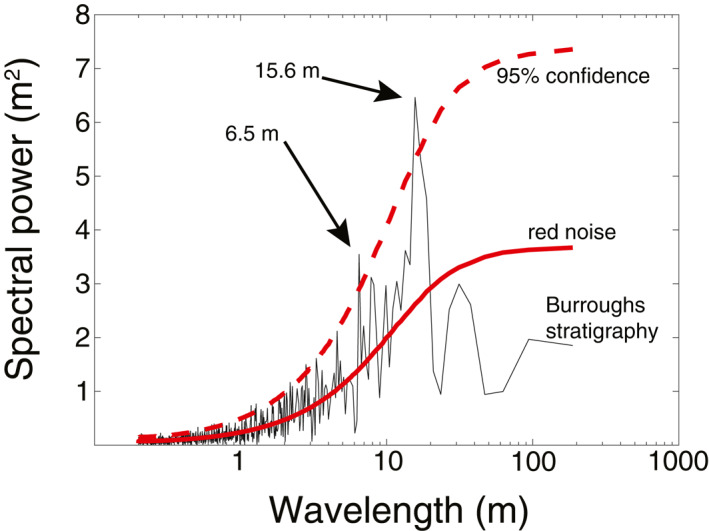
Identification of orbital forcing. Spectral power (black line) as a function of wavelength for the average protrusion profile derived from the digital elevation model in Figure 2a. Average spectral power for a red noise process, calculated from 20,000 randomly generated protrusion profiles with the same mean, standard deviation, and lag‐1 autocorrelation as the real protrusion profile, is shown as a solid red line. The 95% confidence level, calculated from those same 20,000 simulations, is shown as a dashed red line. Spectral power for the real protrusion profile exceeds that of 95% of the randomly generated profiles at wavelengths 6.5 and 15.6 m.

We searched for periodicities in the stratigraphic profiles using Fourier transforms. For the averaged protrusion profile from the DEM with the greatest topographic relief (representing layer exposures with apparent stratigraphic depth 188 m), we found a spectral structure similar to a lag‐1 autoregressive process, a type of red noise relevant in climate records (e.g., Perron & Huybers, 2009). To test the significance of our result, we randomly generated 20,000 synthetic protrusion signals with the same mean, standard deviation, and lag‐1 autocorrelation as the real protrusion profile. We found that two wavelengths in the Fourier power spectrum of the real protrusion profile had power higher than that of 95% of the randomly generated profiles at those wavelengths. Those two wavelengths occur at 15.6 and 6.5 m (Figure 3), with a ratio of 2.4. The other two averaged protrusion profiles, from the other two DEMs, did not display power above the 95% statistically significant threshold, which we interpret as resulting from their smaller stratigraphic section (<100 m).

The 2.4 ratio of dominant wavelengths in the 188‐m‐deep stratigraphy is the same as the ratio between the periodicity (Laskar et al., 2004) of Mars' obliquity changes (∼120 kyr) and that of precession in Mars' argument of perihelion (∼51 kyr), which has been proposed to control recent ice accumulation (Montmessin et al., 2007). This match between ratios is what has previously been argued to be the requirement for confident detection of an orbital signal in Martian ice (Perron & Huybers, 2009). Our detection allows us to invert for an average accumulation rate of the Burroughs crater deposit by interpreting that the 15.6 m wavelength corresponds to a time interval of 120 kyr and the 6.5 m wavelength corresponds to a time interval of 51 kyr. The inferred average accumulation rate is 0.13 mm/yr (“yr” and “Myr” always refer to Earth years in this paper). If the exposed stratigraphy does not contain prolonged (10s of kyr or more) periods of net sublimation such that large parts of the record are missing, then the 188 m of stratigraphy were emplaced over 1.4 Myr. Our inferred accumulation rate is consistent with, but at the lower end of, estimates of the accumulation rate of the SPLD (Becerra et al., 2019), and may suggest lower net accumulation at outlier deposits compared to the PLDs.

The total depth of the Burroughs ice deposit is greater than the 188 m of exposed stratigraphy analyzed here. Topographic analysis (Sori et al., 2019) and depth‐corrected SHARAD radargrams (Figure 3 in Supporting Information S1) both show that the total ice mound thickness is ∼600 m. If the average accumulation rate of the analyzed 188‐m‐thick exposure is representative of the entire stratigraphic column of the Burroughs deposit, then the entire ice deposit was emplaced over 4.5 Myr. If the top of the deposit was emplaced near the present‐day (see Section 4.2) and there are no prolonged episodes of net sublimation (i.e., large missing parts of the record), then this emplacement duration would imply an age of the Burroughs ice deposit of 4.5 Ma. An age of 4.5 Ma would correspond to a large change in average obliquity that occurred (Laskar et al., 2004) between 4 and 5 Ma.

## 4 Discussion

### 4.1 Consistency of the Climate Record in Burroughs

We argue that the local layering, corresponding protrusion profiles, and ensuing Fourier analysis at the sections exposed in HiRISE images are a climate record representative of the entire Burroughs crater deposit for three reasons.

First, individual layers can be visibly traced around the perimeter of the deposit in images that have a lower resolution, but wider footprint, than HiRISE images, like those from MRO's Context Camera (CTX, Malin et al., 2007). Similar procedures have shown the consistency of the climate record expressed in layer protrusion at the NPLD (Becerra et al., 2016).

Second, we found layers throughout the interior of the deposit in radar sounding data. We used MRO's Shallow Radar (SHARAD, Seu et al., 2007) instrument to search for horizontal subsurface reflectors within the Burroughs deposit. Reflected power may come from not only the surface, but also any subsurface interfaces, like internal layers. We analyzed SHARAD tracks that passed through the Burroughs ice deposit for evidence of widespread horizontal reflectors, and found several such reflectors (see Figure 3 in Supporting Information S1). To determine if these found reflectors represent internal layering, we must reject the alternative hypothesis that they are caused by “clutter”. Clutter is power reflected from off‐nadir topography that arrives at similar delay times as it would if it were reflected from a subsurface interface, and thus could mimic internal ice deposit structure. We conducted clutter simulations (Choudhary et al., 2016) using topographic data that yielded artificial radargrams of expected power return from off‐nadir surface topography. This technique is commonly used on Earth (Holt et al., 2006) and Mars (Picardi et al., 2005). We found several internal reflectors in Burroughs that were not predicted in the clutter simulations. We interpreted this result to mean that the reflectors likely represent real internal layering, and that visibly exposed layers are not just features at the surface but extend throughout the deposit and are representative of the Burroughs ice stratigraphy as a whole, similar to what has been observed at the Korolev crater ice deposit in the northern hemisphere (Brothers & Holt, 2016).

Third, we used a tuning procedure called dynamic time warping to tune the three protrusion profiles from the three Burroughs DEMs to each other, and found that the three profiles matched with statistical significance. We used dynamic time warping to quantitatively determine the likelihood that different pieces of stratigraphy represent the same climate record. Dynamic time warping is a signal matching technique first developed for speech pattern recognition but more generally used to compare two time series with uncertainties in their time dimension (Sakoe & Chiba, 1978). This tuning procedure has been applied to terrestrial paleoclimate records (Haam & Huybers, 2010) and has been validated for use in Martian ice stratigraphy (Sori et al., 2014). We used dynamic time warping to tune each of our three stratigraphic columns of Burroughs protrusion to one another. Each tuning yields a cross correlation. For each tuning, we also tuned the Burroughs stratigraphy to 1,000 randomly generated pieces of synthetic protrusion that had the same mean, standard deviation, and lag‐1 autocorrelation as the real stratigraphy. We noted the cross correlation for all 1,000 cases. If the cross correlation in the tuning between real pieces of stratigraphy was greater than the tuning in 90% or more of the random cases, we considered the match significant. A significant match implies that two columns are stratigraphically similar, possibly because they formed contemporaneously. We found this significance test was met for each tuning of a Burroughs protrusion profile to another Burroughs protrusion profile.

The most direct interpretation of the (a) consistency of visible layers around the perimeter of the deposit in CTX images, (b) the detection of internal layering in SHARAD radargrams, and (c) the significant matches between different pieces of Burroughs stratigraphy quantified by dynamic time warping is that local visible layer exposures and the subsequent paleoclimate analysis on them are representative of the Burroughs ice deposit as a whole.

### 4.2 Comparison With the PLDs

Why were we able to identify a ratio of periodicities that exactly matched orbital periodicities in the Burroughs ice deposit, when similar studies of the PLDs (Becerra et al., 2017, 2019) have yielded more complex orbital forcing scenarios? To test for a possible connection between Burroughs and the SPLD, we used tuning to compare the three Burroughs protrusion profiles to protrusion profiles previously derived (Becerra et al., 2019) at 16 different sites throughout the SPLD. For the tuning, we used the same dynamic time warping procedure (Sori et al., 2014) described in Section 4.1, but the two signals being compared are now a protrusion profile from Burroughs and a protrusion profile from the SPLD, rather than two Burroughs profiles to each other.

We found that a statistically significant match does not exist between visible layers at Burroughs crater and layers exposed in the SPLD, using the same 90% threshold described in Section 4.1. Our favored interpretation of these results is that the climate record of Burroughs and the SPLD do not overlap in time. The SPLD surface age has been reported to be at least 10 Ma (Herkenhoff & Plaut, 2000) or 30 Ma, and may be as old as 100 Ma (Koutnik et al., 2002). Some nuance to these ages is warranted because the SPLD is likely composed of units that represent different periods of accumulation (Kolb & Tanaka, 2006; Milkovich & Plaut, 2008), potentially including a younger layer at some locations (Smith et al., 2016; Whitten & Campbell, 2018), but the estimates do imply an age of order 10s of Myrs for the bulk of the SPLD.

To constrain the surface age of the Burroughs ice deposit, we analyzed the size‐frequency distribution of superposed craters. We searched THEMIS and CTX images and determined there are no craters with diameter 300 m or greater superposed on the Burroughs ice deposit. Inferred cumulative cratering rates (Hartmann, 2005) at this size are 3.483 × 10^−11^ yr^−1^ km^−2^, and the ice deposit has total surface area approximately 4,300 km^2^. The probability *p* that a surface area *A* which experiences cratering rate *N* does not form any craters over a time *t* is given by:

(1) t=ln(1/p)/NA

Using this equation, we concluded that the Burroughs surface age is less than 30 Ma with 99% confidence, less than 20 Ma with 95% confidence, and less than 10 Ma with 80% confidence. These age estimates represent crater retention ages. Based on these constraints, it is likely that the entirety of the 4.5 Myr emplacement duration for the Burroughs deposit has occurred since the formation of most of the SPLD.

We propose that a hypothesis advanced by Becerra et al. (2017) is correct: That strongly variable ice accumulation rates lead to alterations of the ratio of orbital periodicities in the PLDs. Complex ice accumulation rates recorded in PLD stratigraphy could result from their deposition over long timespans, especially for the SPLD, or from geographically heterogenous accumulation and sublimation over their great areal extent (e.g., Brown et al., 2016). The Burroughs ice mound was emplaced over a timespan at least several times lower than the SPLD was and is much smaller than the SPLD, and thus may record a climate with less overall variation in accumulation rates than the thicker and larger SPLD. This hypothesis is supported by the observation (Becerra et al., 2019) that some stratigraphic subsections of the Promethei Lingula region of the SPLD display similar power spectra. Alternatively, the regional‐scale climate of crater ice deposits may be fundamentally different than at the PLDs, perhaps with crater topography having important influence on ice accumulation or preservation that leads to a different relationship between orbital changes and climate record.

## 5 Conclusions

We conclude that an ice deposit in Burroughs crater reveals that recent Martian climate has been strongly orbitally controlled. The average accumulation rate at Burroughs has been 0.13 mm/yr, which implies that the visibly exposed stratigraphy formed over 1.4 Myr if no major hiatuses are present in the record. If the exposed stratigraphy is representative of the entire Burroughs deposit, then the ice formed over 4.5 Myr. Finally, if the most recent layer formed near the present‐day as suggested by the superposed cratering record, then the age of the deposit is about 4.5 Ma, which corresponds to a large change in Mars' obliquity. Ultimately, confirmation of absolute ages will require sample analysis either in situ or via sample return, but our results at least show that the onset of ice formation is consistent with strong control by obliquity variation at around 4–5 Ma.

Our work shows that non‐PLD ice deposits on Mars represent valuable, readable records of orbitally forced paleoclimate. In particular, south polar climate over the last few Myrs may be recorded most effectively not in the current SPLD, but in separate crater ice deposits. We thus argue that the crater ice deposits are particularly well suited for studying the climate of the late Amazonian. We speculate that lower latitude deposits like that in Burroughs are more readily deposited and removed during orbital cycles compared to the NPLD and SPLD, as non‐PLD reservoirs may act as fundamentally different sources and sinks of ice and dust than the PLDs (Levrard et al., 2007). Although Burroughs Crater contains the only outlying crater ice deposit with substantial exposed layering appropriate for paleoclimate analysis of topography, future studies could attempt to read the record of subsurface layers observed in SHARAD data at other outlier icy deposits (McGlasson et al., 2021). On Pluto, small crater ice deposits separated from the primary ∼1000‐km‐diameter ice sheet by 100s of km, similar to the distance between Burroughs crater and the SPLD, are observed (White et al., 2017). We suggest that future studies consider high‐resolution imaging or radar sounding of outlier ice deposits on any planet they are found on as a method of obtaining potentially valuable climate records.

## Supporting information

Supporting Information S1

## Data Availability

Figures and the data used in our analysis are additionally available online at https://figshare.com/articles/journal_contribution/Sori_et_al_GRL_2022_data/19204875

## References

[grl63791-bib-0001] Baggenstos, D. , Severinghaus, J. P. , Mulvaney, R. , McConnell, J. R. , Sigl, M. , Maselli, O. , et al. (2018). A horizontal ice core from Taylor Glacier, its implications for Antarctic climate history, and an improved Taylor Dome ice core time scale. Paleoceanography and Paleoclimatology, 33, 778–794. 10.1029/2017pa003297

[grl63791-bib-0002] Bapst, J. , Byrne, S. , & Brown, A. J. (2018). On the icy edge at Louth and Korolev craters. Icarus, 308, 15–26. 10.1016/j.icarus.2017.10.004

[grl63791-bib-0003] Becerra, P. , Byrne, S. , Sori, M. M. , Sutton, S. , & Herkenhoff, K. E. (2016). Stratigraphy of the north polar layered deposits of Mars from high‐resolution topography. Journal of Geophysical Research: Planets, 121, 1445–1471. 10.1002/2015je004992

[grl63791-bib-0005] Becerra, P. , Sori, M. M. , & Byrne, S. (2017). Signals of astronomical climate forcing in the exposure topography of the north polar layered deposits of Mars. Geophysical Research Letters, 44, 62–70. 10.1002/2016gl071197

[grl63791-bib-0004] Becerra, P. , Sori, M. M. , Thomas, N. , Pommerol, A. (2019). Timescales of the climate record in the south polar ice cap of Mars. Geophysical Research Letters, 46, 7268–7277. 10.1029/2019gl083588

[grl63791-bib-0006] Brothers, T. C. , & Holt, J. W. (2016). Three‐dimensional structure and origin of a 1.8 km thick ice dome within Korolev Crater, Mars. Geophysical Research Letters, 42, 1443–1449. 10.1002/2015gl066440

[grl63791-bib-0007] Brown, A. J. , Byrne, S. , Tornabene, L. L. , & Roush, T. (2008). Louth crater: Evolution of a layered water ice mound. Icarus, 196, 433–445. 10.1016/j.icarus.2007.11.023

[grl63791-bib-0008] Brown, A. J. , Calvin, W. M. , Becerra, P. , & Byrne, S. (2016). Martian north polar cap summer water cycle. Icarus, 277, 401–415. 10.1016/j.icarus.2016.05.007

[grl63791-bib-0009] Byrne, S. (2009). The polar deposits of Mars. Annual Review of Earth and Planetary Sciences, 37, 535–560. 10.1146/annurev.earth.031208.100101

[grl63791-bib-0010] Choudhary, P. , Holt, J. W. , & Kempf, S. D. (2016). Surface clutter and echo location analysis for the interpretation of SHARAD data from Mars. IEEE Geoscience and Remote Sensing Letters, 13, 1285–1289. 10.1109/lgrs.2016.2581799

[grl63791-bib-0011] Conway, S. J. , Hovius, N. , Barnie, T. , Besserer, J. , Mouélic, S. , Orosei, R. , AnneRead, N. (2012). Climate‐driven deposition of water ice and the formation of mounds in craters in Mars’ North Polar Region. Icarus, 220, 174–193. 10.1016/j.icarus.2012.04.021

[grl63791-bib-0012] Cutts, J. A. , & Lewis, B. H. (1982). Models of climate cycles recorded in Martian polar layered deposits. Icarus, 50, 216–244. 10.1016/0019-1035(82)90124-5

[grl63791-bib-0013] Haam, E. , & Huybers, P. (2010). A test for the presence of covariance between time‐uncertain series of data with application to the Dongge Cave speleothem and atmospheric radiocarbon records. Paleoceanography, 25, PA2209. 10.1029/2008pa001713

[grl63791-bib-0014] Hartmann, W. K. (2005). Martian cratering 8: Isochron refinement and the chronology of Mars. Icarus, 174, 294–320. 10.1016/j.icarus.2004.11.023

[grl63791-bib-0015] Hays, J. D. , Imbrie, J. , & Shackleton, N. J. (1976). Variations in the Earth’s orbit: Pacemaker of the ice ages. Science, 194, 1121–1132. 10.1126/science.194.4270.1121 17790893

[grl63791-bib-0016] Herkenhoff, K. E. , Byrne, S. , Russell, P. S. , Fishbaugh, K. E. , & McEwen, A. S. (2007). Meter‐scale morphology of the north polar region of Mars. Science, 317, 1711–1715. 10.1126/science.1143544 17885127

[grl63791-bib-0017] Herkenhoff, K. E. , & Plaut, J. J. (2000). Surface ages and resurfacing rates of the polar layered deposits on Mars. Icarus, 144, 243–253. 10.1006/icar.1999.6287

[grl63791-bib-0018] Holt, J. W. , Peters, M. E. , Kempf, S. D. , Morse, D. L. , & Blankenship, D. D. (2006). Echo source discrimination in single‐pass airborne radar sounding data from the Dry Valleys, Antarctica: Implications for orbital sounding of Mars. Journal of Geophysical Research, 111, E06S24. 10.1029/2005je002525

[grl63791-bib-0019] Hvidberg, C. S. , Fishbaugh, K. E. , Winstrup, M. , Svensson, A. , Byrne, S. , & Herkenhoff, K. E. (2012). Reading the climate record of the Martian polar layered deposits. Icarus, 221, 405–419. 10.1016/j.icarus.2012.08.009

[grl63791-bib-0020] Kolb, E. J. , & Tanaka, K. L. (2006). Accumulation and erosion of south polar layered deposits in the Promethei Lingula region, Planum Australe, Mars. International Journal of Mars Science and Exploration, 2, 1–9. 10.1555/mars.2006.0001

[grl63791-bib-0021] Koutnik, M. , Byrne, S. , & Murray, B. (2002). South polar layered deposits of Mars: The cratering record. Journal of Geophysical Research: Planets, 107, E115100. 10.1029/2001je001805

[grl63791-bib-0022] Laskar, J. , Correia, A. C. , Gastineau, M. , Joutel, F. , Levrard, B. , & Robutel, P. (2004). Long term evolution and chaotic diffusion of the insolation quantities of Mars. Icarus, 170, 343–364. 10.1016/j.icarus.2004.04.005

[grl63791-bib-0023] Laskar, J. , Levrard, B. , & Mustard, J. F. (2002). Orbital forcing of the Martian polar layered deposits. Nature, 419, 375–377. 10.1038/nature01066 12353029

[grl63791-bib-0024] Levrard, B. , Forget, F. , Montmessin, F. , & Laskar, J. (2007). Recent formation and evolution of northern Martian polar layered deposits as inferred from a Global Climate Model. Journal of Geophysical Research, 112, E06012. 10.1029/2006je002772

[grl63791-bib-0025] Lewis, K. W. , Aharonson, O. , Grotzinger, J. P. , Kirk, R. L. , McEwen, A. S. , & Suer, T. (2008). Quasi‐periodic bedding in the sedimentary rock record of Mars. Science, 322, 1532–1535. 10.1126/science.1161870 19056983

[grl63791-bib-0026] Malin, M. C. , Bell, J. F. , Cantor, B. A. , Caplinger, M. A. , Calvin, W. M. , Clancy, R. T. , et al. (2007). Context camera investigation on board the Mars reconnaissance orbiter. Journal of Geophysical Research: Planets, 112, E05S04. 10.1029/2006je002808

[grl63791-bib-0027] McEwen, A. S. , Eliason, E. M. , Bergstrom, J. W. , Bridges, N. T. , Hansen, C. J. , Delamere, W. A. , et al. (2007). Mars reconnaissance orbiter’s high resolution imaging science experiment (HiRISE). Journal of Geophysical Research, 112, E05S02. 10.1029/2005je002605

[grl63791-bib-0028] McGlasson, R. A. , Bramson, A. M. , Morgan, G. A. , & Sori, M. M. (2021). Subsurface radar observations of outlier polar ice deposits on Mars. Lunar and Planetary Science Conference, 52, 1649.

[grl63791-bib-0029] Milkovich, S. M. , & Plaut, J. J. (2008). Martian south polar layered deposit stratigraphy and implications for accumulation history. Journal of Geophysical Research: Planets, 113, E06007. 10.1029/2007je002987

[grl63791-bib-0030] Montmessin, F. , Haberle, R. M. , Forget, F. , Langevin, Y. , Clancy, R. T. , & Bibring, J.‐P. (2007). On the origin of perennial water ice at the south pole of Mars: A precession‐controlled mechanism? Journal of Geophysical Research, 112, E08S17. 10.1029/2007je002902

[grl63791-bib-0031] Murray, B. C. , Ward, W. R. , & Yeung, S. C. (1973). Periodic insolation variations on Mars. Science, 180, 638–640. 10.1126/science.180.4086.638 17774288

[grl63791-bib-0032] Perron, J. T. , & Huybers, P. (2009). Is there an orbital signal in the polar layered deposits on Mars? Geology, 37, 155–158. 10.1130/g25143a.1

[grl63791-bib-0033] Picardi, G. , Plaut, J. J. , Biccari, D. , Bombaci, O. , Calabrese, D. , Cartacci, M. , Cicchetti, A. , et al. (2005). Radar soundings of the subsurface of Mars. Science, 310, 1925–1928. 10.1126/science.1122165 16319122

[grl63791-bib-0034] Sakoe, H. , & Chiba, S. (1978). Dynamic programming algorithm optimization for spoken word recognition. IEEE Transactions on Acoustics, Speech, & Signal Processing, 26, 43–49. 10.1109/tassp.1978.1163055

[grl63791-bib-0035] Schaefer, H. , Petrenko, V. V. , Brook, E. J. , Severinghaus, J. P. , Reeh, N. , Melton, J. R. , Mitchell, E. (2009). Ice stratigraphy at the Pâkistoq ice margin, West Greenland, derived from gas records. Journal of Glaciology, 55, 411–421. 10.3189/002214309788816704

[grl63791-bib-0036] Seu, R. , Phillips, R. J. , Biccari, D. , Orosei, R. , Masdea, A. , Picardi, G. , et al. (2007). SHARAD sounding radar on the Mars reconnaissance orbiter. Journal of Geophysical Research: Planets, 112, E09003. 10.1029/2006je002745

[grl63791-bib-0037] Smith, I. B. , Haynec, P. O. , Byrned, S. , Becerrae, P. , Kahref, M. , Calving, W. , et al. (2020). The Holy Grail: A roadmap for unlocking the climate record stored within Mars’ polar layered deposits. Planetary and Space Science, 184, 104841. 10.1016/j.pss.2020.104841

[grl63791-bib-0038] Smith, I. B. , Putzig, N. E. , Holt, J. W. , & Phillips, R. J. (2016). An ice age recorded in the polar deposits of Mars. Science, 352, 1075–1078. 10.1126/science.aad6968 27230372

[grl63791-bib-0039] Sori, M. M. , Bapst, J. , Becerra, P. , & Byrne, S. (2019). Islands of ice on Mars and pluto. Journal of Geophysical Research: Planets, 124, 2522–2542. 10.1029/2018je005861

[grl63791-bib-0040] Sori, M. M. , Perron, J. T. , Huybers, P. , & Aharonson, O. (2014). A procedure for testing the significance of orbital tuning of the Martian polar layered deposits. Icarus, 235, 136–146. 10.1016/j.icarus.2014.03.009

[grl63791-bib-0041] White, O. L. , Moore, J. M. , Mckinnon, W. , Spencer, J. R. , Howard, A. D. , Schenk, P. M. , et al. (2017). Geological mapping of sputnik planitia on pluto. Icarus, 287, 261–286. 10.1016/j.icarus.2017.01.011

[grl63791-bib-0042] Whitten, J. L. , & Campbell, B. A. (2018). Lateral continuity of layering in the Mars south polar layered deposits from SHARAD sounding data. Journal of Geophysical Research: Planets, 123, 1541–1554. 10.1029/2018je005578

