# Supplementary material for: Orbital Forcing of Martian Climate Revealed in a South Polar Outlier Ice Deposit
Source: Geophys Res Lett. 2022 Mar 29;49(6):e2021GL097450. doi: 10.1029/2021GL097450 (PMC9285921; doi:10.1029/2021GL097450)
Supplement: Supplementary file 1 — Supporting Information S1 [file GRL-49-0-s001.pdf]

Geophysical Research Letters

Supporting Information for

Orbital forcing of Martian climate revealed in a south polar outlier ice deposit

Michael M. Sori[1], Patricio Becerra[2], Jonathan Bapst[3], Shane Byrne[4], and Riley A. McGlasson[1]

[1]Department of Earth, Atmospheric, and Planetary Sciences, Purdue University, West Lafayette, IN 47907, USA.
[2]Physikalisches Institut, Universität Bern, Bern, Switzerland.
[3]Jet Propulsion Laboratory, California Institute of Technology, Pasadena, CA 91190, USA.
[4]Lunar and Planetary Laboratory, University of Arizona, Tucson, AZ 85716, USA.

Contents of this file

Figure S1
Figure S2
Figure S3

Introduction

This Supporting Information provides additional details. It provides examples of layered ice deposits that are insufficient for paleoclimate analysis (Figure S1), examples of adjacent topographic profiles space 5 meters apart in the Burroughs ice mound, and SHARAD analysis of the ice deposit in Burroughs crater (Figure S3).

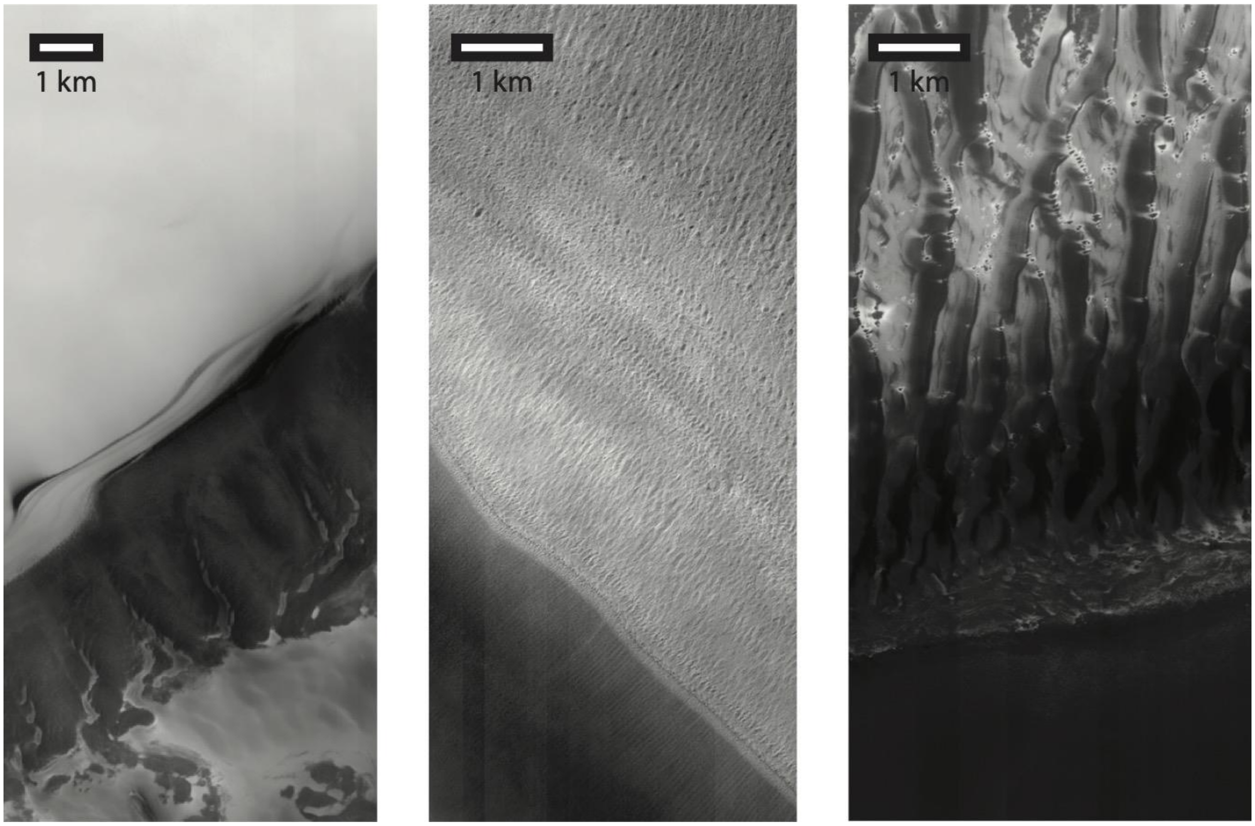

Supplementary Figure 1. Examples of obscured ice mounds. HiRISE images ESP_054968_2575 (left), ESP_056975_1070 (middle), and ESP_056896_1075 (right) show ice mounds at Dokka Crater in the north polar region, an unnamed crater at 73.1°S, 181.9°E in the south polar region, and Richardson Crater in the south polar region. All three ice mounds show evidence for some degree of layering, but layer exposures are either extremely limited in lateral and stratigraphic extent (Dokka), obscured by surface texture (unnamed crater), or obscured by heavy cover of superposed dunes (Richardson). This obfuscation contrasts with the extensive layer exposures seen at Burroughs Crater in Figure 1 of the main text.

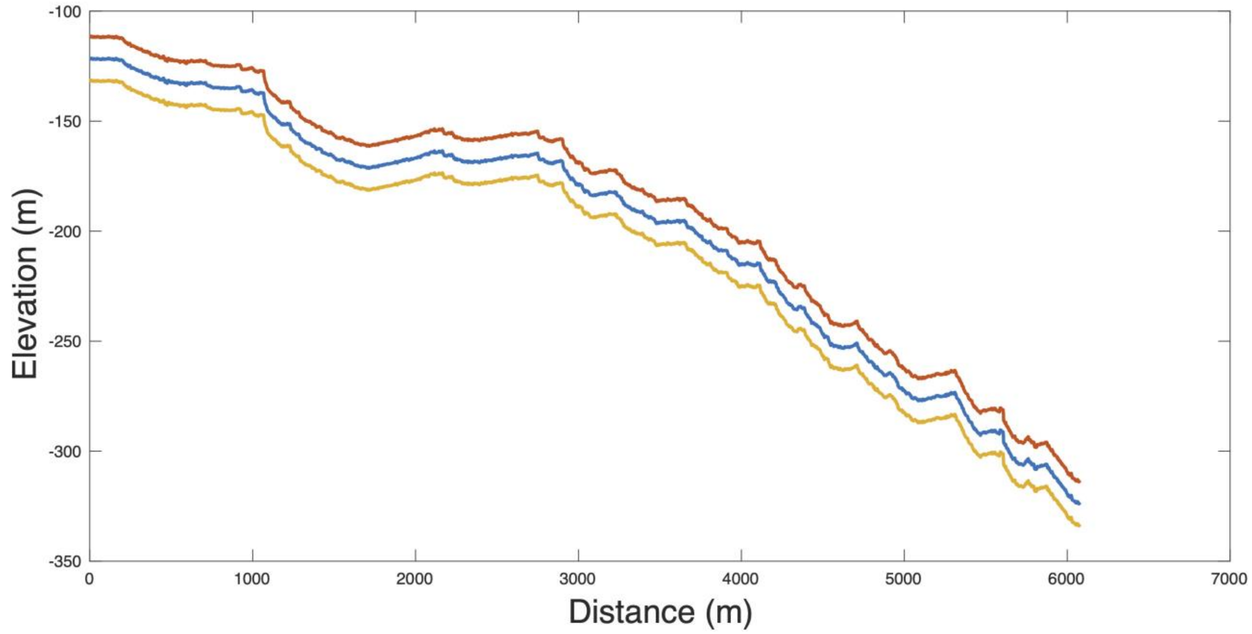

Supplementary Figure 2. Three topographic profiles spaced 5 meters apart extracted from DEM DTEPC_058362_1070_057650_1070_A01, showing high similarity between nearby profiles. The red and yellow profiles have 10 meters added and subtracted to elevation for visualization purposes.

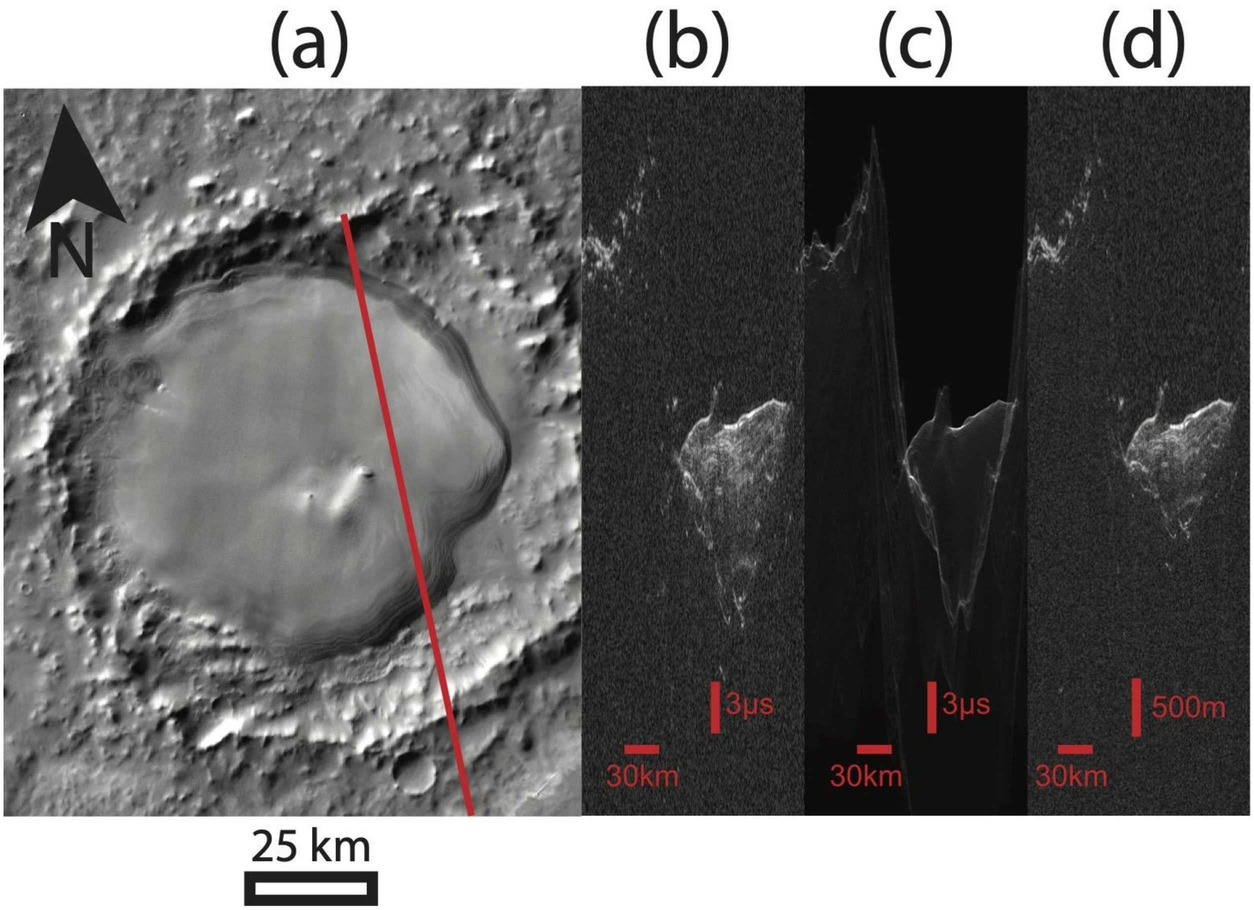

Supplementary Figure 3. Radar analysis of Burroughs crater. (a) THEMIS daytime infrared image of Burroughs crater, with red line representing SHARAD track s_00230702. (b) Time delay radargram of portion of SHARAD track s_00230702 represented by red line in (a). (c) Clutter simulations of SHARAD track S_00230702 showing the expected radar return from regional topography. (d) Depth-corrected radargram of SHARAD track s_00230702, assuming a real component of the dielectric permittivity of 3.15. Note that there is horizontal layering visible in (b) and (d) that is not predicted in the clutter simulation in (c).
